# Geometric properties of nucleic acids with potential for autobuilding

**DOI:** 10.1107/S0108767310039140

**Published:** 2010-11-11

**Authors:** Tim Gruene, George M. Sheldrick

**Affiliations:** a Department of Structural Chemistry, Georg-August-University Göttingen, Tammanstrasse 4, D-37077 Göttingen, Germany

**Keywords:** nucleic acids, autobuilding, geometric properties, electron-density distribution

## Abstract

Medium- to high-resolution X-ray structures of DNA and RNA molecules were investigated to find geometric properties useful for automated model building in crystallographic electron-density maps. We describe a simple method, starting from a list of electron-density ‘blobs’, for identifying backbone phosphates and nucleic acid bases based on properties of the local electron-density distribution. This knowledge should be useful for the automated building of nucleic acid models into electron-density maps. We show that the distances and angles involving C1′ and the P atoms, using the pseudo-torsion angles  and  that describe the …P—C1′—P—C1′… chain, provide a promising basis for building the nucleic acid polymer. These quantities show reasonably narrow distributions with asymmetry that should allow the direction of the phosphate backbone to be established.

## Abstract 2

Algorithms and geometrical properties are described for the automated building of nucleic acids in experimental electron density.

## 1. Introduction

In macromolecular crystallography the construction of an initial model has been greatly facilitated by programs that automatically build a model into an experimental electron-density map given a protein sequence (Morris *et al.*, 2002 ▶; Terwilliger, 2000 ▶; Cowtan, 2006 ▶; Emsley & Cowtan, 2004 ▶; Emsley *et al.*, 2010 ▶). Recently some programs have extended these capabilities to nucleic acid structures (Pavelcik & Schneider, 2008 ▶; Hattne & Lamzin, 2008 ▶; Terwilliger *et al.*, 2008 ▶), but in general the automated tracing of nucleic acids is less highly developed than for proteins.

In this paper a simple but fast procedure to place phosphates and bases in electron density is presented and some geometrical features of nucleic acid structures that might be useful for automated tracing are explored. This should enable the development of algorithms for tracing nucleic acids in experimental electron-density maps that are relatively efficient and robust, whilst accommodating most conformations that are found in practice. The description of DNA and RNA geometry in this paper is of necessity much less comprehensive than in previous surveys based on, for example, seven-dimensional conformational space spanned by the seven backbone angles α–ζ (Murray *et al.*, 2003 ▶; Saenger, 1989 ▶). The validity of such simplified representations has been discussed by other authors (Duarte & Pyle, 1998 ▶; Wadley *et al.*, 2007 ▶).

After we had completed our work on the location of phosphates and bases, and convinced ourselves of the advantages of constructing the backbone based on …P—C1′—P—C1′… chains (as discussed below), a paper appeared by Keating & Pyle (2010 ▶) on RNA model building that was also based on the C1′ approach. Their method makes sophisticated use of RNA rotamer libraries but requires the user to provide the phosphate and base coordinates and hence complements our work. Since these authors plan to make their software available as a plug-in to the program *COOT* (Emsley *et al.*, 2010 ▶; Emsley & Cowtan, 2004 ▶), we plan to do the same now, starting with the automated phosphate and base location, even though the optimization of our proposed strategy for building a complete RNA or DNA model is likely to take several years. We believe that our work at the present stage will make a valuable contribution and that it might be more effective for the crystallographic community if we make our algorithms and source code available at this stage. The C++ sources will be made available on TG’s web site (http://shelx.uni-ac.gwdg.de/~tg) when this paper is published. This should facilitate the complete semi-automated building of RNA structures in the experimental electron density. Although our C1′-approach results could be regarded as simply a confirmation of Keating and Pyle’s work, we show here that this approach is advantageous for DNA as well as for RNA structures.

## 2. Algorithms

### 2.1. Test data sets

Table 1 ▶ describes the test data sets used for assessing the quality of the phosphate- and the base-localizing algorithms described in this section. For all test sets except octan, phases were calculated from coordinates and experimental intensities. Experimental phases for octan were determined by single-wavelength anomalous diffraction (SAD) from the Br atoms in bromouracil that had been substituted for thymine. Using calculated phases was necessary in the other cases because experimental phases for structures containing nucleic acids are almost impossible to obtain.

### 2.2. Detection of bases and phosphates

#### 2.2.1. ‘Blob’ search

Since a simple peak search would not be appropriate for finding the bases (at high resolution the density in the middle of a pyrimidine ring should be a local minimum, not a maximum), the following algorithm has been devised to locate ‘blobs’ of electron density in the map that might correspond to phosphates or nucleotide bases. The initial density is first ‘normalized’ by dividing it by its standard deviation determined over the full map. Experience showed that this simple step makes it easier to establish default settings that are independent of the data set and to minimize the required user input. Especially in the presence of brominated uracil (as in the test set octan) it turned out to be useful to modify the electron density further by 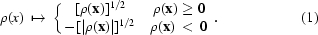

               

This is the same procedure as used in *SHELXE* (Sheldrick, 2010 ▶) for protein backbone tracing. A masking map is constructed on the same grid as the input map with all grid-point values set to 1 to initially mark all voxels (volume increments, one per grid point) as valid. The individual voxels of the original map are then sorted in descending order of their normalized density values. Iterating through this sorted list starting with the highest density, a voxel is only considered if the value of its grid point in the masking map is 1. A sphere of 2.5 Å is constructed around the selected voxel, and the centre of gravity 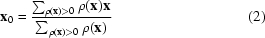
of the normalized density is calculated in this sphere. The centre of the sphere is moved to this point and the procedure repeated until the shift is less than a preset cutoff (0.11 Å). If this does not happen within 20 iterations, or if the distance to the initial grid point is greater than 3.5 Å, or if the value of the masking map at the converged centre is −1, the peak is rejected. Otherwise, the ‘blob’ centre 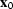 is accepted and the grid points of the masking map that are within 1.3 Å of the converged centre are set to −1.

In order to select base planes and phosphates, first the ‘moments of density’ 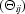 are calculated within the sphere of 2.5 Å about the converged centre 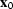. These are similar to the moments of inertia of a rigid body:
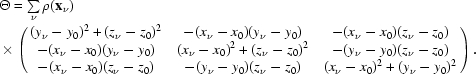

               

The sum in 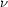 ranges over all *positive* voxels within 2.5 Å of 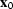. The eigenvalues 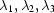 and corresponding eigenvectors 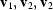 of this symmetric matrix 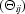 are calculated and used to select phosphates and bases from the blob list as described below. The eigenvalues and the corresponding eigenvectors of this positive-definite matrix are sorted such that 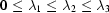.

#### 2.2.2. Detection of bases

The density of a base has a flat shape even at medium resolution. In terms of the ‘moments of density’, this is reflected by one large eigenvalue with its eigenvector perpendicular to the plane of the base and two smaller eigenvalues with eigenvectors parallel to the base plane. A second property of a base is its large overall density. Bases can be detected based on these two properties by sorting the blob list by the quantity 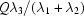. *Q* is the sum of the electron density of all voxels with positive density within the sphere of radius 2.5 Å. Following the detection of the base plane, one standard purine and one standard pyrimidine (Parkinson *et al.*, 1996 ▶) each are aligned to the plane and then fitted to the density in two steps.

In step 1, the standard base is rotated in steps of 15° in the base plane. At each step the fit is optimized approximately by the simplex method. The target function is the negative correlation coefficient between the atomic numbers of the atoms of the rigid body and the modified electron density at the corresponding positions. In order to improve the plane stability and to increase the contrast between purines and pyrimidines, dummy atoms are included in the models at positions where no density is expected and are used during the simplex minimization with an atomic number of −1 in the calculation of the correlation coefficient between electron-density value and atomic number. A similar simplex procedure is employed in *SHELXE* for protein backbone extension.

In step 2, at each ‘blob’ a cluster of the top six solutions of both the purine and the pyrimidine models are stored and then further refined by maximizing the target function 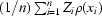, where 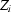 is the atomic number of the *i*th atom in the base and 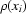 is the modified experimental electron density at the position of this atom in the map. The minimization uses the vector Broyden–Fletcher–Goldfarb–Shanno (BFGS) algorithm as implemented in the GNU Scientific Library (Galassi *et al.*, 2009 ▶). The dummy atoms are not included in this step.

**Figure 1:**
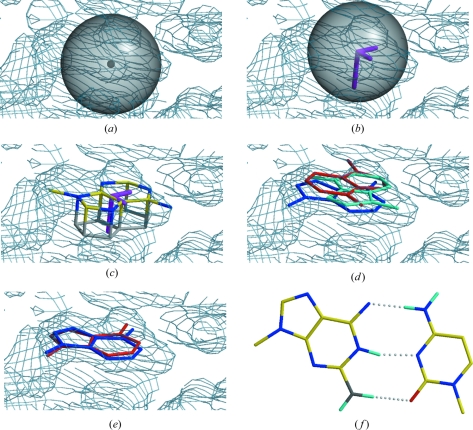
Progress of fitting a ‘blob’ to the centre of the base. (*a*) ‘Blob’ selected after sorting the map voxels together with the 2.5 Å sphere used to find the centre of the base. (*b*) The sphere after convergence of the ‘centre of density’ together with the eigenvectors of the matrix 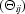. The length of each ‘vector’ corresponds to the value of its eigenvalue. (*c*) Search base (here a purine) aligned to the eigenvectors within the base plane. (*d*) Top three purines after simplex fitting. The *CC* values used for sorting are 0.992 (blue), 0.989 (cyan) and 0.988 (red). (*e*) The best fitting purine before (blue) and after (red) the gradient fitting. (*f*) The selected base with its Watson–Crick partner and the hydrogen bonding used for scoring.

Subsequently the potential purines and pyrimidines for each cluster are tested for Watson–Crick base pairing. H atoms are attached to the purine and the pyrimidine as shown in cyan in Fig. 1 ▶(*f*). The scoring function is the sum of the cosines of the three N—H⋯*A* angles and the moduli of the deviations of the three H⋯*A* hydrogen bonds from 2.16 Å for terminal N atoms or from 1.85 Å for other N atoms as hydrogen-bond donors. These mean distances were derived from a search of the Cambridge Structural Database (Allen, 2002 ▶). Since in most structures a large fraction of bases is involved in base pairing, this provides a powerful and independent selection criterion, and for those clusters from step 2 for which a Watson–Crick base pair can be detected, the remaining (false) bases can be pruned. A future development could include base pairs or triplets other than the standard Watson–Crick pairs. An example of the progress of convergence of the 2.5 Å sphere and subsequent fitting of the base can be seen in Fig. 1 ▶. The results of this search procedure for the test data sets are shown in Table 2 ▶.

In cases where a (partial) protein model is already available, *e.g.* after running *SHELXE* (Sheldrick, 2010 ▶), *ARP/WARP* (Morris *et al.*, 2002 ▶) or *PHENIX* (Terwilliger *et al.*, 2008 ▶) for a nucleic acid/protein complex, the protein region can be masked out. This was easily accomplished with a few lines of code with the help of the Clipper C++ library (Cowtan, 2003 ▶).

#### 2.2.3. Detection of phosphates

Phosphates possess two distinctive features that should help to identify them in the blob list: strong and tetrahedrally shaped density. The three eigenvalues of the density in the sphere enclosing the corresponding blob (see above) should be similar. In addition, the tetrahedrally shaped density should manifest itself as a negative correlation coefficient between voxels related by inversion through the P atom. For the purpose of locating phosphates, a copy of the blob list is sorted in descending order of the function: 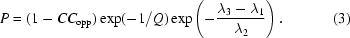

               

*Q* is defined as above. 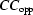 is the correlation coefficient of the electron-density values at diametrically opposite points on a sphere of radius 1.56 Å about the centre of the blob, *i.e.* the putative P-atom position. For sufficiently tetrahedrally shaped density at high resolution 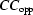 should approach −1, maximizing the factor 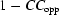 in equation (3). Using the expression 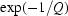 instead of *Q* was found to reduce the number of false positives caused by heavy atoms such as metal cations. The last exponential term has its maximum when the minimum and the max­imum eigenvalues are equal. As a result of the exponential functions, *P* has a maximum of 2, independent of the input map. The quality of this scoring function is summarized in Table 3 ▶ where 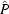 is the top score, 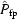 is the score of the first false positive, 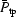 is the average score of all true positives and 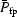 is the average score of all false positives up to the last true positive (and therefore not available for those cases in which there are no false positives amongst the true positives). The quality indicator r.n. is defined as the ratio between *the number of blobs at the top of the sorted list needed to include the given fraction of correct phosphates* and *the number of phosphates corresponding to the given fraction*. It is 1 in the absence of false positives and becomes larger the more false positives are present.

### 2.3. Backbone tracing

Once phosphates and bases are placed, the backbone must be traced. This means that the putative phosphates and bases must be correctly aligned in space and that false positives must be excluded. What appears to be easy for the crystallographer is difficult to automate.

Duarte & Pyle (1998 ▶) described the conformation of the RNA backbone based on the 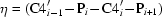 and 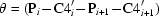 pseudo-torsion angles, analogous to the 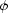,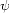-Ramachandran plot for proteins (Ramachandran *et al.*, 1963 ▶). The methods described above to locate phosphates and bases in electron-density maps are effective even at medium resolution. Relative to a fixed base, the C1′ atom has very little freedom of movement. Since the sugar ring tends to be rather flexible and less well described in the density, we replace C4′ with C1′ in the above definitions of 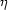 and 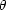, and use the angles 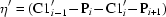 and 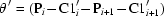 to facilitate the construction of the correct backbone from the list of putative phosphates and bases (Gruene, 2008 ▶; Keating & Pyle, 2010 ▶). Several groups have investigated the use of preferred conformations of RNA (*i.e.* rotamers) for backbone building (Keating & Pyle, 2010 ▶; Pavelcik & Vanco, 2006 ▶; Pavelcik & Schneider, 2008 ▶; Schneider *et al.*, 2004 ▶). While at lower resolution this is probably the only possible approach, this work focuses on a slightly different approach with more localized and possibly less bias-prone properties. Therefore, in addition to 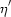 and 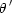, the following quantities involving the phosphates and C1′ atoms were evaluated for their usefulness for autotracing:

(i) distances 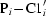 and 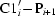, and
(ii) angles 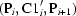 and 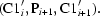

The inclusion of more distant atoms only results in broader distributions that are less useful for autotracing.

## 3. Distance and angle statistics from the NDB

The structures used to accumulate the statistical distributions employed in the proposed tracing algorithm were extracted from the Nucleic Acid Database (NDB; Berman *et al.*, 1992 ▶) by selecting crystallographic data with a resolution of 1.5 Å or better and structures containing either RNA only or DNA only. By July 2010, the NDB contained 53 structures of DNA in the *A* conformation, 79 structures of DNA in the *B* conformation, 31 structures of DNA in the *Z* conformation, and 61 RNA structures that met these criteria. RNA was not split into *A*, *B* and *Z* conformations because too few database structures are in the *B* conformation or the *Z* conformation. The desired distances and angles were calculated using the program *nanalysis* written in C++ for this purpose. The program and its source code can be obtained *via* TG’s web site (http://shelx.uni-ac.gwdg.de/~tg).

The geometrical data were further processed with the program *R* (R Development Core Team, 2010 ▶). The mean angle and its resultant length of angular data were calculated as
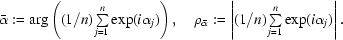
For *Z*-DNA, for which all distributions show two peaks, the data sets were split into two sets in order to maximize the weighted sum of 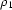 and 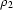. For the angle (C1′, P, C1′), this was the case at 81.37°, leaving the first data set with 151 values and the second one with 87 values. For the angle (P, C1′, P), this was the case at 80.50°, leaving the first data set with 155 values and the second one with 83 values.

**Figure 2:**
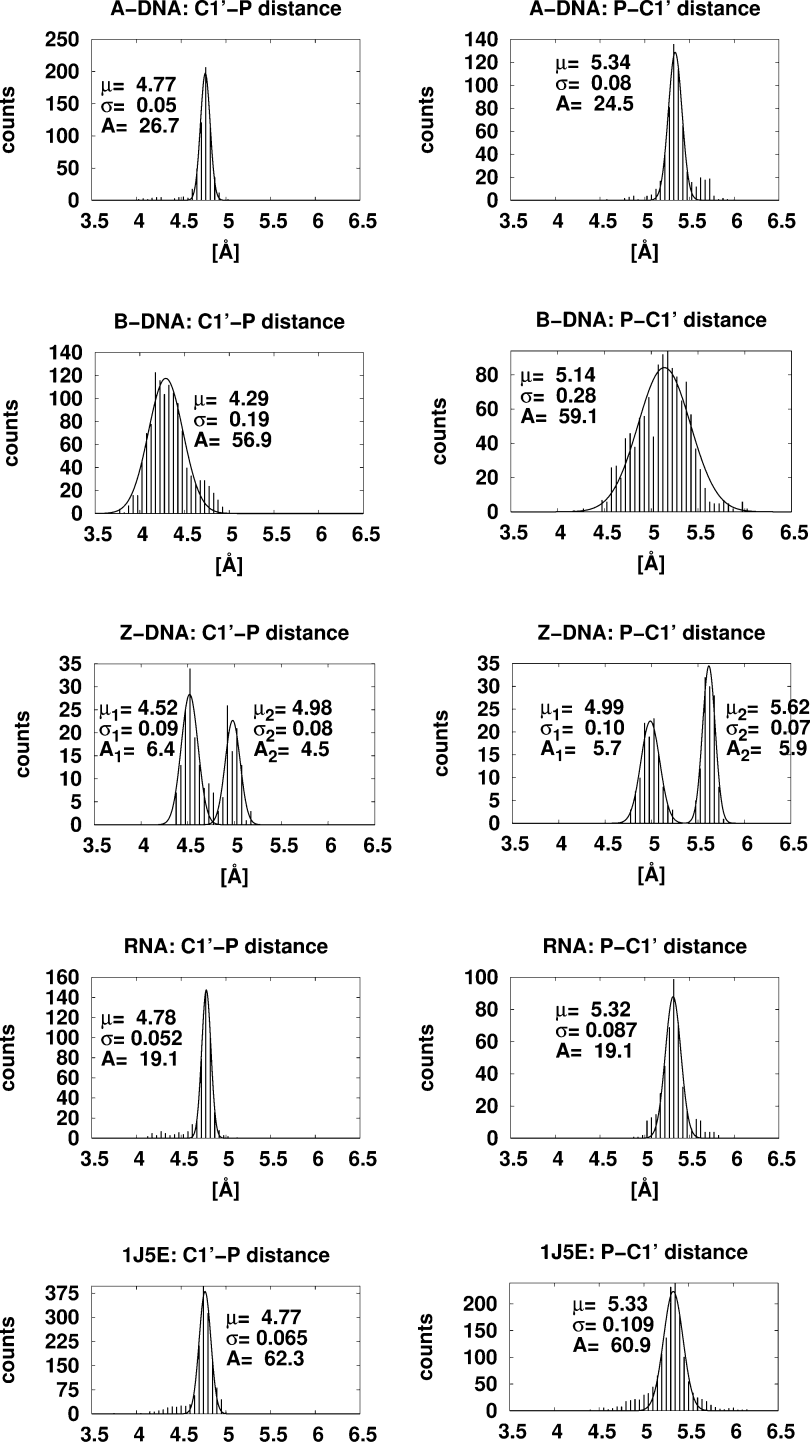
Histograms of the C1′
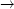
3′P (left) and 5′P
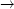
C1′ (right) distance distributions for RNA and DNA and the fitted normal distribution 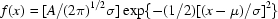 corresponding to Table 4 ▶. See §3 for more details. 1J5E is the PDB code of the 30S ribosomal subunit determined at 3.05 Å resolution and shown for comparison with the RNA histograms.

In order to determine the mean and standard deviations for the distances, their histogram data were fitted to normal distributions 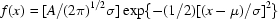 using the Marquardt–Levenberg algorithm as implemented in *gnuplot* (Williams *et al.*, 2010 ▶). In the case of *Z*-DNA the distributions show two peaks and the sum of two normal distributions was fitted. The resulting mean values and standard deviations are summarized in Table 4 ▶. The distance distributions are shown in Fig. 2 ▶ and the angular distributions are shown in Fig. 1 of the supplementary material.^1^

## 4. Results and discussion

This work presents efforts to develop algorithms to build atomic models automatically into the crystallographic electron-density map of nucleic acids. The procedure is separated into two parts: First putative backbone P atoms and bases are located in the modified electron-density map, then they are assembled into a strand and the missing atoms are filled in. The first part has been implemented in the program *KNUSPR*. For the second part we propose an algorithm based on local properties of nucleic acids presented in this work.

### 4.1. Localization of phosphates and bases

Section 2 describes the localization of phosphates and bases based on a ‘blob’ search. A ‘blob’ is a spherical region of the electron-density map with a radius of 2.5 Å. A ‘blob’ with this radius does not cover purines, but it avoids overlap with adjacent bases. From the density distribution inside each ‘blob’, using criteria as described in §2.2, the list of blobs is subdivided into a list of tetrahedrally shaped phosphate ‘blobs’ and a list of flat base ‘blobs’.

The program *KNUSPR* was written to apply the sorting functions to experimental electron-density maps at various resolutions and the resulting lists were compared with the refined structures for these maps in order to assess the reliability of the selection criteria. The reference structures that were used for this purpose are listed in Table 1 ▶.

Tables 2 ▶ and 3 ▶ summarize the success rate for the base and phosphate searches, respectively, in comparison with the refined coordinates using various test data sets. A phosphate ‘blob’ was counted as correct if it was within 1.5 Å of a P atom in the reference structure. A base was considered to be correctly placed if the C1′ position was less than 1 Å from the correct position and the root-mean-square deviation of atoms common to purines (C1′, C2, C4, C5, C6, N1, N3, O2) or to pyrimidines (C1′, C2, C4, C5, C6, C8, N1, N3, N7, N9) was less than 1.0 Å. These criteria are stricter than normally employed and would probably need to be relaxed for low resolution.

A relative quality indicator r.n. is defined in §2.2.3 as the *relative number* of blobs at the top of the sorted list that are required to cover a certain percentage of correct phosphates. It is always greater than or equal to 1.0 and the closer it approaches 1.0 the better the selection. In all reference structures all phosphates were found in the blob list with 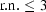. Even for the ribosome structure (PDB code 1J5E, using experimental amplitudes and phases to 3.05 Å resolution and masking out the protein), 1455 out of 1513 phosphates (96%) are correctly found with an overall r.n. = 4.4 (details not shown). The more concentrated the true phosphate and base positions are at the top of the two ‘blob’ lists, the smaller the number of permutations required to build contiguous backbone chains will be.

The success rate for the base search algorithm is displayed in Table 2 ▶. Resolution is a more limiting factor for bases than for phosphates: for the ribosome structure only 6% of all bases could be located, possibly because the density of the bases loses its flatness with decreasing resolution (data not shown in Table 2 ▶).

### 4.2. Criteria for backbone tracing

Once putative phosphates and bases are placed in the electron-density map, the next step is to find the correct order to build the nucleic acid backbone, *i.e.* one starts from a base C1′ atom and identifies from the phosphate candidates the correct two phosphates upstream and downstream that match the electron density. From these, the best matching bases are found *etc*. This procedure includes separating true positives from false ones in the two lists of phosphate and base candidates.

The conformation of nucleic acids consists of seven torsion angles α–ζ (Saenger, 1989 ▶). This compares with only two angles 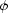 and 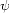, usually displayed as Ramachandran plot, that are sufficient to describe the secondary structure of proteins. Some simplifications are clearly necessary to reduce the computational complexity. In order to describe the backbone configuration of RNA, Duarte & Pyle (1998 ▶) suggested that it is sufficient to use only two pseudo-torsion angles 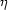 and 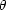 instead of all seven angles α–ζ. This greatly reduces the number of possible conformations that have to be considered in automated model building.

#### 4.2.1. Backbone directionality


**Figure 3:**
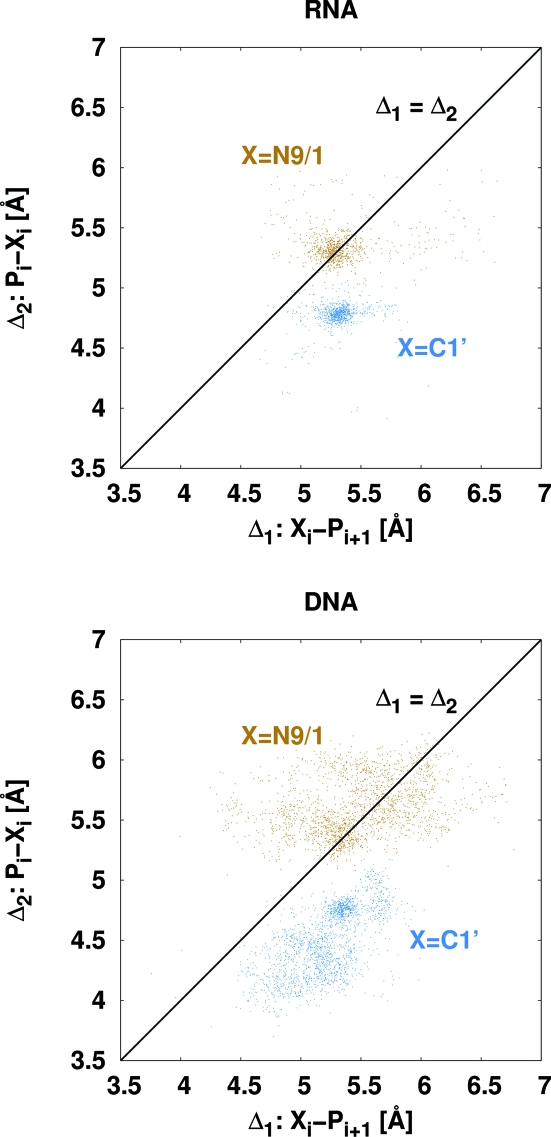
The scatter plots of the distances 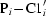 *versus* 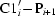 (blue) show a clear asymmetry allowing determination of the direction of the backbone chain. The brown points show the distances 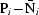 *versus* 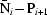 where 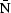 is N9 for purines and N1 for pyrimidines. The asymmetry is absent for these 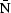-based data points.

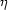
 and 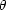 are based on the P and C4′ atoms. The ideas presented above, however, place phosphates and bases. With a fixed base and P atom, the position of the C4′ atom is not well defined because of the flexibility of the sugar ring. This led to the idea of replacing the C4′ atom in this model with one of the base atoms, preferably one that is present in all bases. Suitable candidates are C1′ and N9 (purines) or N1 (pyrimidines). The statistics are very similar for both cases. Fig. 3 ▶ shows the distribution of the distance from C1′ and N9/N1 to the next base’s phosphate *versus* the distance of phosphate to C1′ and N9/N1, respectively, within the same base. The distance distribution to the upstream and downstream P atom is asymmetric with respect to C1′, but rather symmetric with respect to N9/N1. Therefore, choosing C1′ as an anchor point over N9/N1 provides additional information about the directionality of the nucleic acid backbone. For this reason all subsequent statistics are based on the C1′ atom instead of N9/N1. The corresponding angles 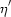 and 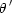 were named in analogy with 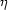 and 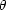 (Duarte & Pyle, 1998 ▶). Although we were not aware of the work on RNA tracing by Keating & Pyle (2010 ▶) until after their paper appeared, they fortunately adopted the same definitions of 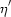 and 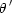.

The 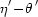 plot does not differ significantly from the 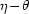 plot (they are compared in Fig. 2 of the supplementary material). Although the analyses of Duarte & Pyle (1998 ▶) and Wadley *et al.* (2007 ▶) were based on RNA only, this plot shows that the distribution for DNA provides an equally valuable guide for building backbone chains of alternating P and C1′ atoms. In neither case are these distributions sufficient, and further criteria are required for correct and reliable automated model building.

Table 4 ▶ shows four additional quantities that are potentially useful for chain tracing. These quantities are the C1′ to 3′P distance, the 5′P to C1′ distance and the angles (C1′—P—C1′) and (P—C1′—P). The NDB holds enough DNA structures to distinguish between the *A*, *B* and *Z* conformations. There are only one *B* conformation and five *Z* conformations for RNA with resolution better than 1.5 Å, so for RNA all data were consolidated.

Table 4 ▶ summarizes the mean values and standard deviations. In practice the errors in the C1′ positions obtained by fitting the bases are likely to be greater than these relatively small standard deviations except at high resolution, and these C1′ errors would introduce correlations, *e.g.* between the two P—C1′ distances that have a C1′ atom in common. Despite this, once a backbone chain of alternating phosphates and C1′ atoms has been constructed, the presented distributions and in particular the plot of the 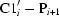 distance against the 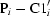 distance shown in Fig. 3 ▶ show a clear asymmetry that should allow the reliable determination of the direction of the backbone trace when the full chain is taken into account.

## 5. Conclusions

In this article a program is presented that places phosphates and nucleic acid bases into crystallographic electron-density maps. The algorithms work rather well for phosphates even for experimental phases of complicated structures like the ribosome (PDB code 1J5E) at 3.05 Å resolution. For bases the useful resolution limit is currently about 2.5 Å.

Based on the P and C1′ atom positions from the placed phosphates and bases, angle and distance distributions are presented in Table 4 ▶ that should then allow the building of chains of alternating P and C1′ atoms and even assignment of the directionality of these chains. The chains can be verified by comparing their 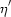 and 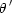 angles with the distributions discussed above. For lower resolution it will almost certainly be necessary to make use of a rotamer library (Keating & Pyle, 2010 ▶; Murray *et al.*, 2003 ▶; Schneider *et al.*, 2004 ▶). Finally, the more flexible sugars that are usually less clearly defined in the density could be inserted between the P and C1′ atoms and the geometry globally optimized to fit the experimental density with the help of appropriate distance, angle, torsion angle and planarity restraints.

We intend to make our work available both as a stand-alone program and in the form of a plug-in for the interactive graphics program *COOT* (Emsley *et al.*, 2010 ▶), starting with the phosphate and base location, where it should prove complementary to the RNA tracing plug-in planned by Keating & Pyle (2010 ▶), which requires the user to specify the positions of the phosphate and the base atoms.

## Supplementary Material

Supplementary material file. DOI: 10.1107/S0108767310039140/sc5036sup1.pdf

## Figures and Tables

**Table 1:** Details of the test data sets used for assessing the quality of phosphate and base search algorithms. Phases were calculated from coordinates and experimental data except for octan, for which bromouracil SAD data were available

ID	Reference	Description	Resolution (Å)	No. of bases
egli1	Bancroft *et al.* (1994 ▶)	*Z*-DNA	1.01	12
egli2	Tereshko *et al.* (1999 ▶)	*B*-DNA	1.12	24
octan	Shakked *et al.* (1983 ▶)	*A*-DNA	1.70	16
udo	Heinemann & Hahn (1992 ▶)	*B*-DNA	1.76	20
419D	Shi *et al.* (2000 ▶)	DNA/RNA hybrid	2.20	32
2HOJ	Edwards & Ferré-D’Amaré (2006 ▶)	RNA	2.50	78
3AFA	Tachiwana *et al.* (2010 ▶)	DNA/protein	2.50	292

**Table 2:** Evaluation of the base search algorithm #B is the total number of bases and #bp the number of Watson–Crick base pairs in the reference structure. The average r.m.s.d. (for true positives only) refers to the atoms (C1′, C2, C4, C5, C6, N1, N3, O2) for pyrimidines and to (C1′, C2, C4, C5, C6, C8, N1, N3, N7, N9) for purines. #tp and #fp are the numbers of true and false positive pairs. The ‘Total’ column counts the true positives from both the paired bases and unpaired bases in the clusters (see §2.2.2), with the percentage of the total number of bases in parentheses.

ID	Resolution (Å)	#B	#bp	#tp	#fp	R.m.s.d.	Total (%)
egli1	1.01	12	6	6	3	0.05	12 (100%)
egli2	1.12	24	12	12	1	0.04	24 (100%)
octan	1.70	16	8	8	5	0.10	16 (100%)
udo	1.76	20	10	10	3	0.20	20 (100%)
419D	2.20	32	12	12	3	0.17	31 (97%)
2HOJ	2.50	78	31	16	9	0.26	65 (83%)
3AFA	2.50	292	146	24	2	0.35	114 (39%)

**Table 3:** Evaluation of the phosphate search algorithm The p% column states at what position in the list of putative phosphates p% of the total number of phosphates were found, *e.g.* the entry 8 in the 80% column of egli1 means that in order to cover eight phosphates (80% of 10), the first eight peaks of the sorted list of phosphate candidates are required. See §2.2.3 for an explanation of the quality indicator r.n and the definition of the last four column labels.

ID	#P	80%	r.n.	90%	r.n.	100%	r.n.	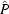	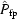	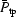	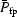
egli1	10	8	1.00	9	1.00	10	1.00	1.19	0.55	1.06	n/a
egli2	22	18	1.00	20	1.00	22	1.00	1.31	0.34	0.70	n/a
octan	14	12	1.00	13	1.00	14	1.00	1.37	0.42	1.18	n/a
udo	18	15	1.00	17	1.00	18	1.00	1.38	0.71	1.15	n/a
419D	28	23	1.00	26	1.00	76	2.71	1.39	0.43	0.92	0.22
2HOJ	78	67	1.06	78	1.10	183	2.35	1.51	0.82	0.94	0.26
3AFA	290	306	1.32	423	1.62	825	2.84	1.45	1.11	0.77	0.30

**Table 4:** Average angles and distances between 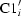 and 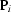, 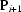, respectively Values were determined as explained in §3. The direction is always 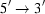.

		C1′—P			P—C1′			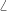 (C1′, P, C1′)			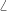 (P, C1′, P)		
	No. of data	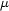 (Å)	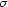 (Å)	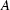 (Å)	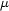 (Å)	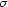 (Å)	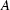 (Å)	No. of data	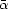 (°)	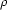	No. of data	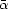 (°)	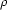
*A*-DNA	737	4.77	0.05	26.7	5.34	0.08	24.5		65.7	0.993		73.2	0.991
*B*-DNA	1171	4.29	0.19	56.9	5.14	0.28	59.1		64.6	0.983		90.6	0.987
*Z*-DNA	238	4.52	0.09	6.4	4.99	0.10	5.7	151	76.1	0.999	155	74.4	0.999
		4.98	0.08	4.5	5.62	0.07	5.9	87	86.4	0.999	83	86.7	0.998
RNA (all)	1318	4.78	0.05	19.1	5.32	0.09	19.1		67.9	0.985		71.5	0.993

## Footnotes

1 Supplementary data for this paper are available from the IUCr electronic archives (Reference: SC5036). Services for accessing these data are described at the back of the journal.
