# Supplementary material for: Geometric properties of nucleic acids with potential for autobuilding
Source: Acta Crystallogr A. 2010 Nov 11;67(Pt 1):1–8. doi: 10.1107/S0108767310039140 (PMC3006036; doi:10.1107/S0108767310039140)
Supplement: Supplementary file 1 [file a-67-00001-sup1.pdf]

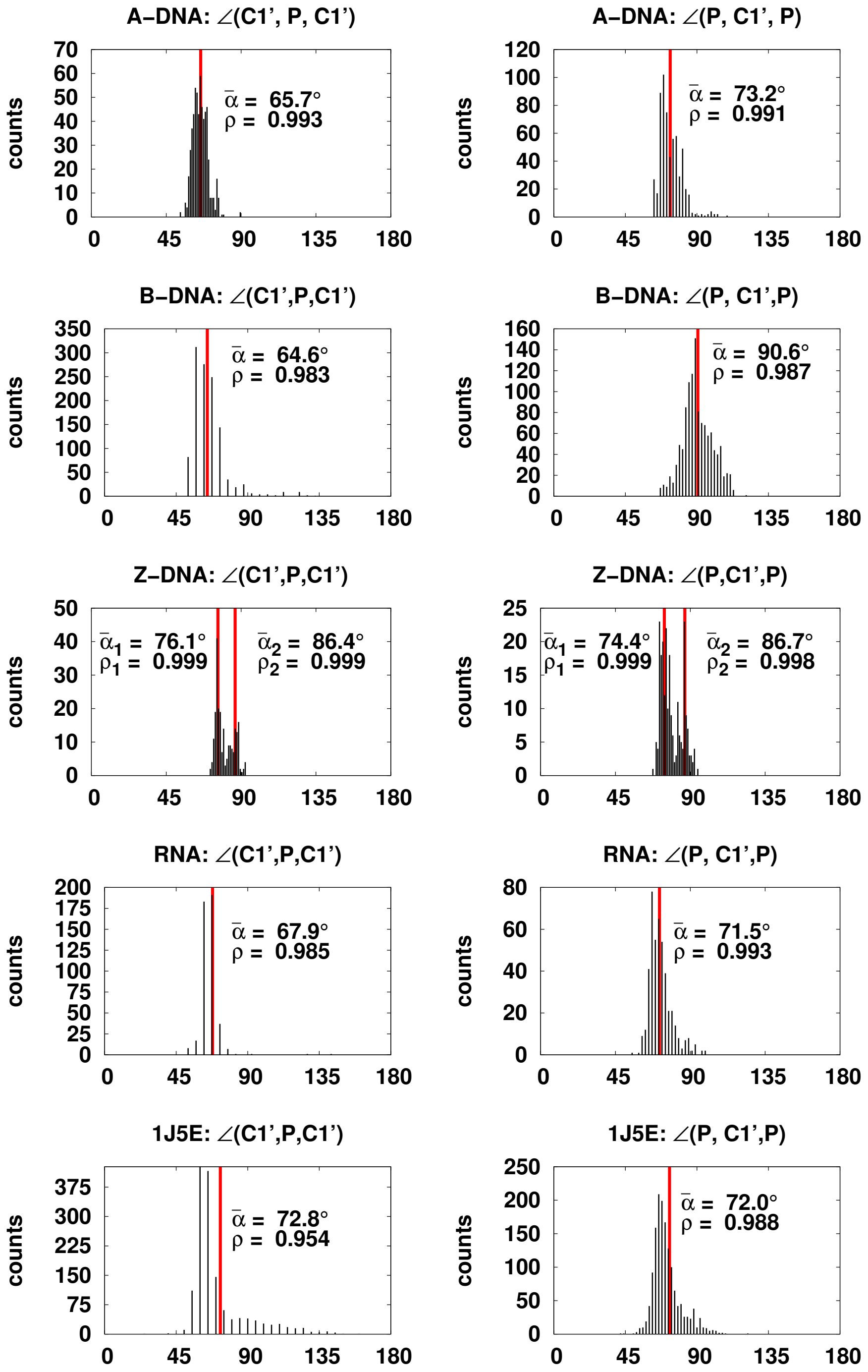

Figure 1: Distribution of the (C1'-P-C1') (left) and (P-C1'-P) (right) angle for RNA and DNA corresponding to Table 4. See section 3 for details about the parameters $\bar{\alpha}$ and ρ. 1J5E is the PDB-ID of the 30S ribosomal subunit determined at 3.05 Å resolution and shown for comparison with the RNA histograms.

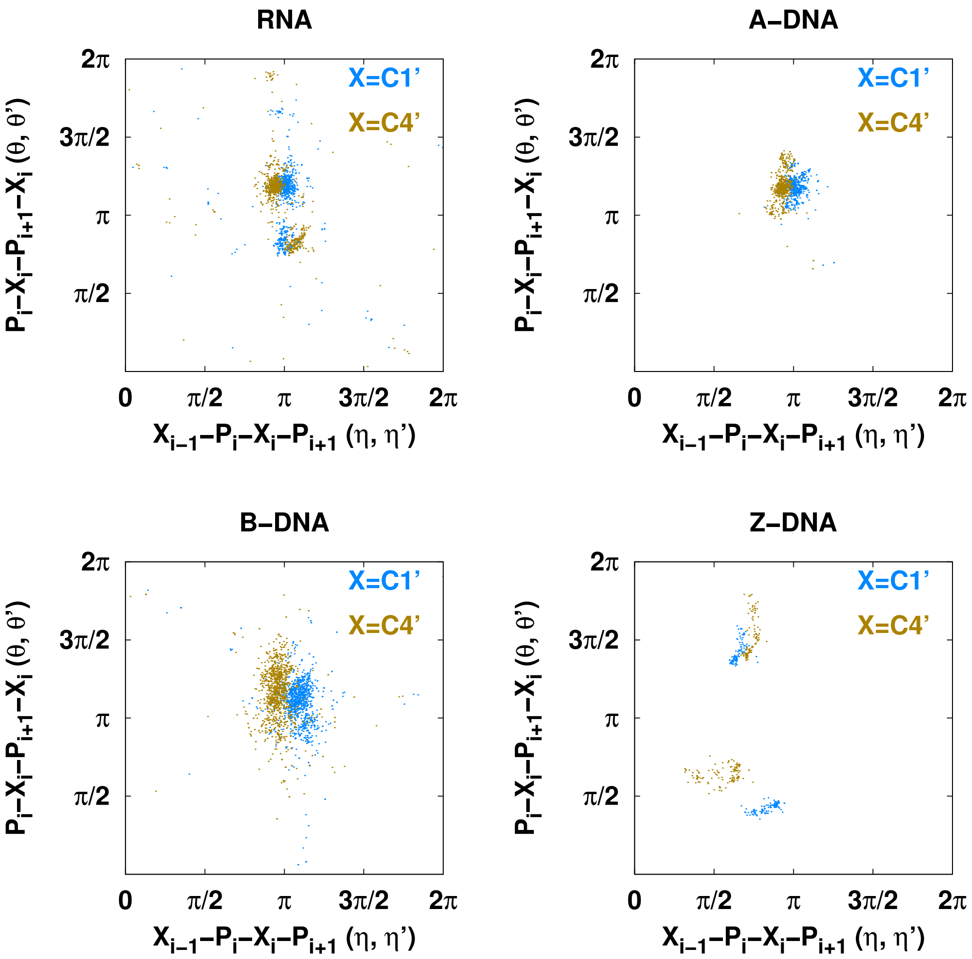

Figure 2: Comparison between the η, θ plot based on C4$'$ (brown) suggested by Wadley *et al.* (2007) and the η', θ' plot based on C1$'$(blue). It can be seen in each plot that the distribution based in C1$'$ is similar to the distribution based on C4$'$, especially with respect to the spread. These distributions can be used to score the quality of a backbone trace.
